# Morphology and Variations of the Posterior Cerebral Artery: A Literature Review

**DOI:** 10.7759/cureus.81205

**Published:** 2025-03-25

**Authors:** Neha Xalxo, Lalit Ratanpara, Krishna S Patil, Pradip R Chauhan, Simmi Mehra

**Affiliations:** 1 Anatomy, All India Institute of Medical Sciences, Rajkot, Rajkot, IND; 2 Anatomy, National Institute of Medical Sciences and Research, Jaipur, Jaipur, IND

**Keywords:** basilar artery, circle of willis, posterior cerebral artery, posterior circulation, posterior communicating artery

## Abstract

The human brain is supplied by a complex network of vessels, including the circle of Willis (CoW), which provides collateral circulation to ensure optimal perfusion during vascular blockages. An essential component of the CoW is the posterior cerebral artery (PCA), which supplies blood to the occipital lobe, visual cortex, and other cortical brain areas. The PCA typically originates from the bifurcation of the basilar artery but can show variations, such as “fetal-type” PCA. These variations, including hypoplasia, aplasia, duplication, and fenestration, can considerably impact cerebral vasculature and increase the risk of ischemic stroke, aneurysms, and visual impairment. The PCA is divided into multiple segments (P1, P2, P3, P4, and sometimes P5), each with diverse anatomical and morphological variations. Variations in PCA morphology can complicate cerebrovascular management and influence surgical approaches. Understanding these variations is crucial for accurate diagnosis and effective treatment of PCA-related complications. This literature review explores the anatomy of the PCA, including its embryological development, the implications of its variations, and the possible clinical outcomes related to these anomalies.

## Introduction and background

The human cerebrum is perfused by myriads of arteries that supply oxygenated blood. The unique cerebral vasculature features a circular ring of anastomosing arteries, known as the circle of Willis (CoW) or circulus arteriosus cerebri, which forms collateral circulation to the brain [1]. This collateral circulation is of crucial advantage, as blood can be rerouted through other arteries within the circle, minimizing the risk of damage and stroke in the case of an arterial blockage [2]. The CoW is completed by the anterior cerebral artery (ACA), the internal carotid artery (ICA) at its distal end, the middle cerebral arteries (MCA), the anterior communicating artery (AComA), the posterior communicating artery (PComA), the posterior cerebral artery (PCA), and the basilar artery (BA) [3]. PComA interconnects both the anterior and posterior circulations of the brain by interlinking the ICAs on either side to the terminal branches of the BA, like the PCA.

In 70% of occurrences, the PCA originates from the bifurcation of the BA, while 20% and 10% arise from the posterior communicating arteries and a combination of the two, respectively [1]. The PCA extends from its commencement toward the occipital region, coursing over the tentorium cerebelli and around the cerebral peduncle. It supplies the occipital lobe, posteromedial temporal lobes, midbrain, thalamus, choroid plexus, and part of the lateral and third ventricles [4].

As the carotid territory supplies blood to a large part of the brain, an unusual origin of the PCA from the ICA may not cause occipital pole infarction in the case of BA thrombosis. However, when the internal carotid or common carotid is ligated during surgery, or in thromboembolism of this artery, the occipital pole becomes vulnerable to infarction [5,6]. The occipital lobe has the primary visual cortex of the human brain. Since the PCA supplies this area, any anatomical variation or damage to this artery can lead to various vision-related pathologies [4].

There are fewer studies on the anatomy of the PCA than the anterior and middle cerebral arteries; however, rare and distinctive anomalies are still being reported. The degree of contribution from the vertebrobasilar or carotid systems to the PCA's origin, as well as other variations, is clinically important, as these can lead to changes in blood flow and potentially contribute to cerebrovascular insufficiency [7-9]. The aim of this literature review is to explore the anatomy and anomalies of the PCA.

## Review

The PCA is an important part of the posterior circulation, with its morphology and anatomical variations extensively studied through cadaveric dissection and imaging modalities. Current literature review provides a comprehensive understanding of the detailed anatomy, variations, and its embryological basis, building upon previous studies.

Divisions of the PCA

The anatomical division of the PCA into segments has been extensively studied both in cadavers and angiographically to better understand its morphology and clinical significance. Several classifications of the PCA segments have been proposed in the literature [10-14]. Traditionally, the PCA is divided into P1, P2, P3, and P4 segments [10,11,15,16], while some authors also include P5 (5th segment) of the PCA [4,17].

**Figure 1:**
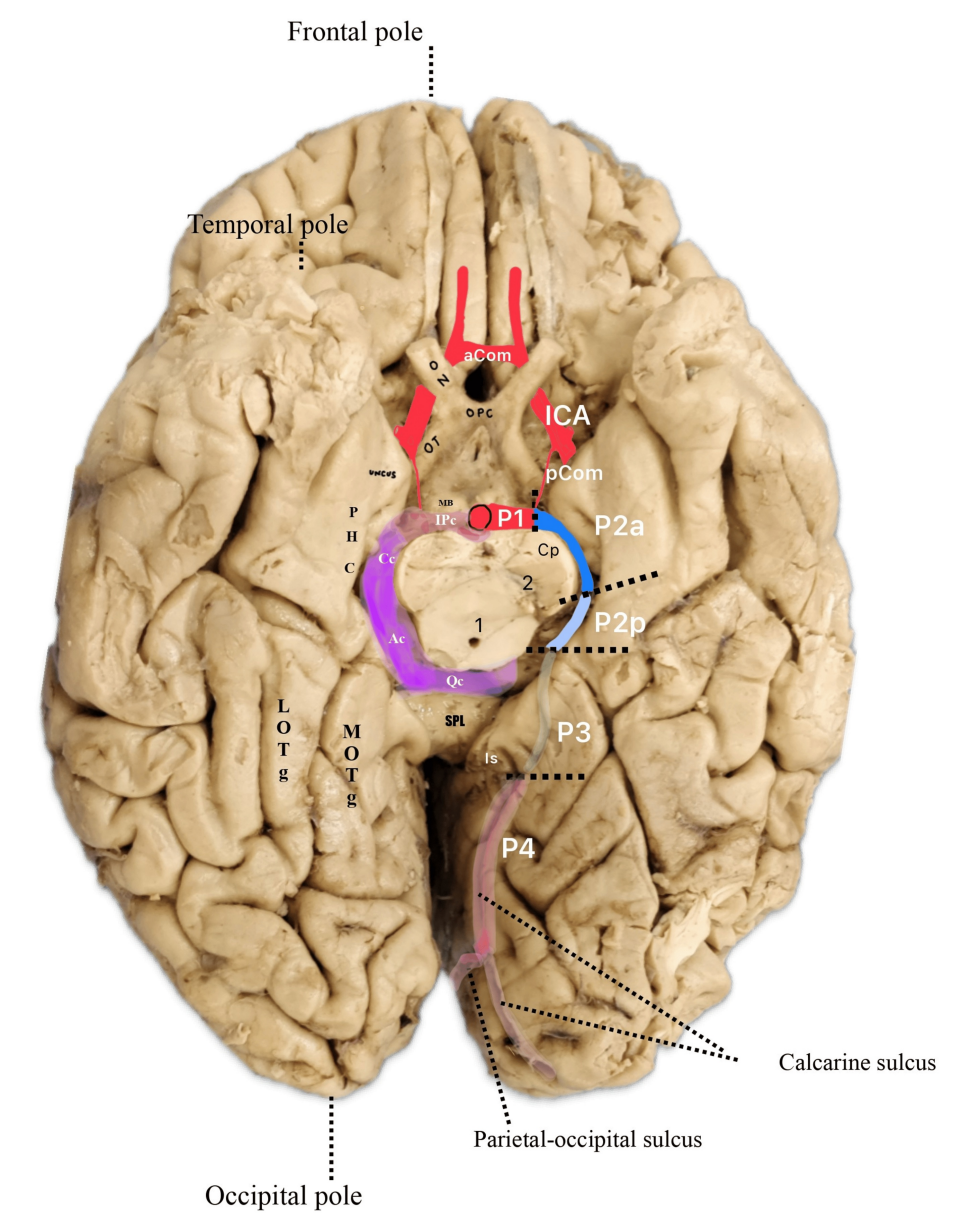
Inferior view of brain, depicting four segments of PCA: P1, P2, P3, and P4 ICA: internal carotid artery; aCom: anterior communicating artery; pCom: posterior communicating artery; ON: optic nerve; OT: optic tract; OPC: optic chiasma; PHC: parahippocampus; LOTg: lateral occipitotemporal gyrus; MOTg: medial occipitotemporal gyrus; MB: mamillary body; Cp: cerebral peduncle; SPL: splenium; Is: isthmus; IPc: interpeduncular cistern; Cs: crural cistern; Ac: ambient cistern; Qc: quadrigeminal cistern; 1: cerebral aqueduct; 2: substantia nigra; P2a: anterior segment of P2; P2p: posterior segment of P2 Image credit: Dr. Neha Xalxo

Krayenbüh et al. in their angiographic study of cerebral arteries describe only two segments of the PCA, circular (basilaris) and cortical segments, with the circular segment corresponding to the current P1 and P2 segments, whereas the cortical segment corresponds to the P3 and P4 segments [10]. Margolis et al. also describe angiographic segments of the PCA: peduncular, ambient, and quadrigeminal cistern segments, which correspond to the anatomic P1, P2, and P3 segments, respectively (Figure 1) [11].

Segments of PCA

P1 segment is also known as precommunicating, proximal, peduncular, mesencephalic circular, basilar, and interpeduncular segment [14,18,19]. This segment extends from the bifurcation of BA to the origin of PcomA within the interpeduncular cistern while passing over the oculomotor nerve (Figure 1) [4,20,21]. P2 segment is also known as post-communicating, distal, and perimesencephalic segment [4,14,18-21].

This segment is divided into two parts: P2a and P2p. The p2a part (anterior/crural) traverses posterolaterally between the cerebral peduncle medially and the uncus laterally within the crural cistern [22,23]. This part is also known as the peduncular or crural segment [18,19,24]. The P2p (posterior) portion begins at the posterior border of the crural cistern, running posteriorly through the ambient cistern (located on the sides of the midbrain at the level of the tentorium cerebelli) [24,25]. This part ends at the lateral edge of the midbrain tectum, where it lies on the superior surface of the parahippocampal gyrus [14,26]. It is also called the ambient segment or lateral mesencephalic segment (Figure 1) [14,18].

The P3 segment, also known as the quadrigeminal segment, extends from the lateral aspect of the quadrigeminal cistern at the origin of the posterior temporal artery to the anterior limit of the calcarine fissure (Figure 1) [24,27].

**Figure 2:**
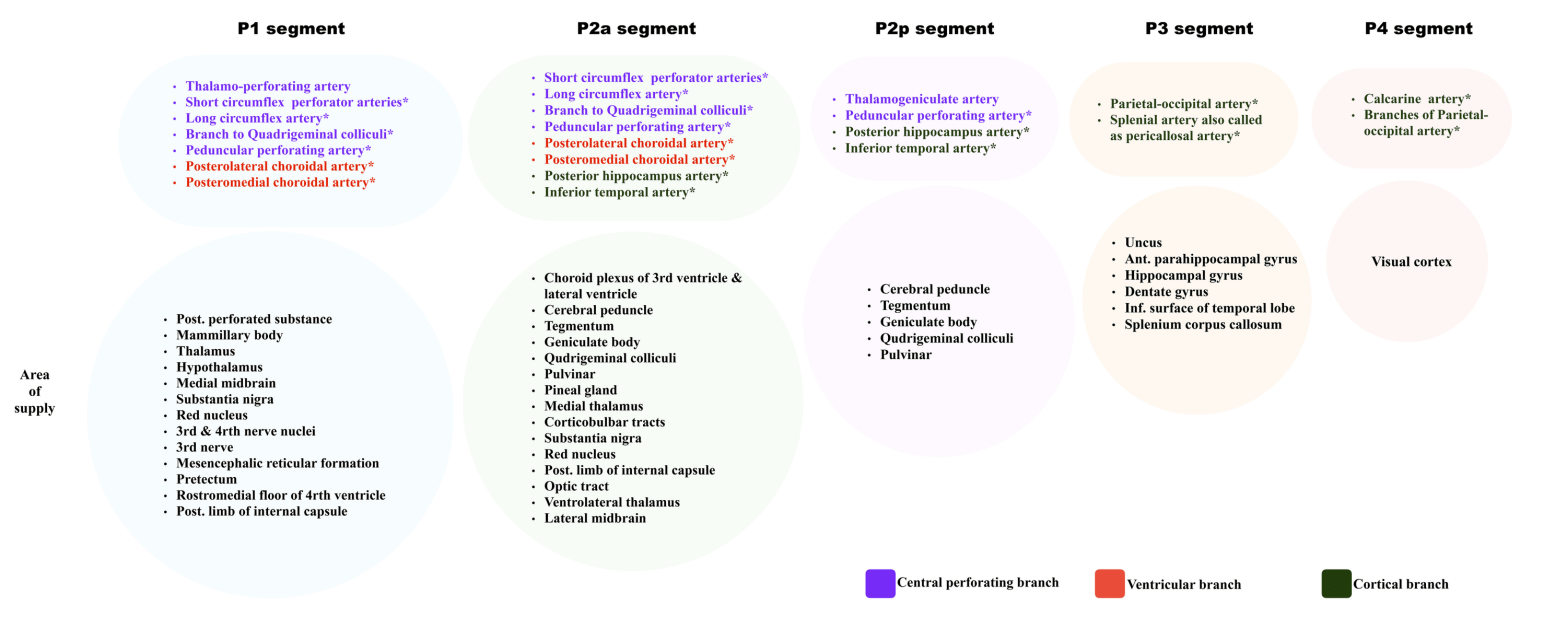
Branches of the PCA segments: P1, P2, P3, and P4 PCA: posterior cerebral artery *Branches with variable origin from different segments Image credit: Dr. Neha Xalxo

The P4 segment/cortical segment runs over the parieto-occipital and calcarine sulcus [4,20]. The P5 segment is also described in literature as the terminal part of the PCA, which includes branches that arise from the parieto-occipital and calcarine arteries (Figure 1) [4,20]. Branches of various segments of PCA and the area of supply are shown in Table 1 (Figure 2) [8,9].

**Table 1:** Branches of the PCA and area of supply PCA: posterior cerebral artery

Type of branch of PCA	Branches	Main areas of supply
Central perforating	Thalamoperforating, thalamogeniculate, peduncular perforating	Diencephalon, midbrain, posterior perforated substance, cerebral peduncle
Ventricular	Posteromedial choroidal artery (PMChA), posterolateral choroidal artery (PLChA)	Choroid plexus (3rd and 4th ventricles), lateral ventricle, temporal horn
Cortical	Parieto-occipital artery, splenial Artery (pericallosal)	Visual cortex, occipital lobe, splenium of corpus callosum

Embryology

The embryogenesis of the CoW transpires in two phases: the preliminary formation of several arterial plexuses and the subsequent regression of specific arterial segments, either in utero or postnatally, leading to the adult configuration [28].

During early embryonic life, the brain's vascular network is rudimentary, enveloped by a thick capillary plexus, except for nonvascular midline strips. Cerebral circulation commences at five weeks of gestation with the emergence of six pairs of branchial arch arteries. The arteries of the third branchial arch participate in the development of the ICA around day 24 of embryogenesis [5,28,29].

The ICA splits into an anterior (rostral) and posterior (caudal) branch by day 28 [4,30]. The rostral branch forms the anterior choroidal artery (AchoA), ACA, and MCA, which supply the cerebrum and upper brainstem. Concurrently, carotid-basilar anastomoses (CBAs) supply the lower brainstem [28]. The caudal branch extends to the midbrain and forms the mesencephalic and diencephalic arteries. It also gives rise to the fetal posterior cerebral artery (fPCA), PcomA, its main branches, the posterior choroidal artery (PchoA), and the future P1 segment of the PCA, which is absent during 5-8 weeks of initial development. The development of the P1 segment begins at nine weeks [28,31].

In the early phase, the hindbrain is supplied by the carotid and vertebrobasilar system, through the trigeminal artery (TA), otic artery (OA), hypoglossal artery (HA), and proatlantal artery (ProA). The TA, OA, and HA undergo regression as the PcomA develops and links to the distal BA, while the ProA persists until the vertebral arteries mature [28,32].

The evolution of the PCA is a dynamic process that transpires across multiple embryonic stages. During the 4th to 5th week, the PCA-related vasculature begins to emerge in the P1 segment. At this stage, AchoA supplies the diencephalon and telencephalon. By the 5th week, the PchoA develops and forms an anastomosis with the AchoA. The mesencephalic and diencephalic arteries play a crucial role in cerebral perfusion. Between five and six weeks, substantial branches of the AchoA extend to the medial wall of the telencephalic vesicle, facilitating the development of the PCA. At six weeks, AchoA ceases its telencephalic supply, and the PchoA develops additional branches that supply the medial telencephalic wall, indicating the first creation of the PCA. The mesencephalic and auxiliary mesencephalic arteries persist in supplying midbrain structures [28].

Around the seventh week, the telencephalic branch of the PchoA gradually expands, eventually becoming the primary supply of the PCA, which later merges into the vertebrobasilar system. Between 12 and 16 weeks, the PCA becomes clearly identifiable. It gives off mesencephalic, diencephalic, and lateral posterior choroidal branches, which ultimately supply the medial occipital lobe. The diencephalic artery stops supplying the telencephalon, possibly due to the brain's caudal enlargement, around 15 to 17 weeks. Around 22 weeks, the P1 segment of the PCA may expand to support the posterior cerebral circulation [28].

**Figure 3:**
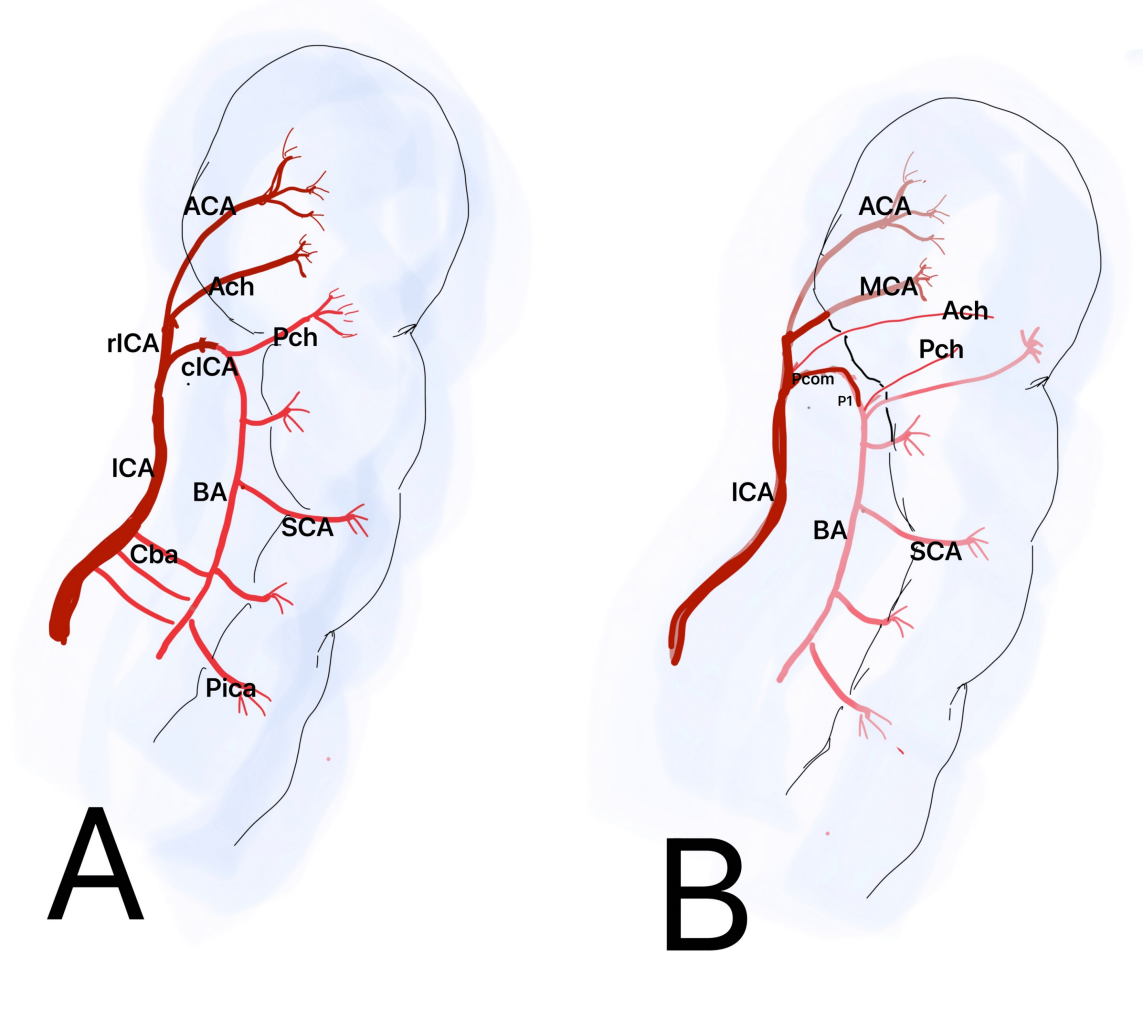
Embryonic development of the PCA PCA: posterior cerebral artery (A) Internal carotid artery (ICA) divides into rostral ICA (rICA) and caudal ICA (cICA), anterior choroidal artery (Ach), posterior choroidal artery (Pch), a branch of the cICA, superior cerebellar artery (SCA), posterior inferior cerebellar artery (Pica), middle cerebral artery (MCA), basilar artery (BA), and carotid basilar anastomosis (Cba). (B) Communicating artery (PcomA) and the P1 segment are developed Image credit: Dr. Neha Xalxo

As the posterior fossa and occipital lobe grow, the posterior circulation becomes independent from the anterior circulation, resulting in the obliteration of anterior-posterior anastomoses. The posterior communicating arteries then serve as the sole adult anastomoses between the anterior and posterior circulations (Figure 3) [30,33].

The adult PCA connects with the BA as branches from the fetal PCA and forms the distal segment of the BA. When the fetal PCA persists and originates from the ICA, it may reduce the caliber of the BA. This occurs in 10%-29% of the population, mostly unilaterally [33]. The persistence of a fetal PCA can be classified as partial or complete, based on the hypoplasia of the P1 segment of the PCA. Patients exhibiting ipsilateral persistence of a prenatal PCA are unable to form leptomeningeal anastomoses among the ACA, MCA, and PCA, rendering them more vulnerable to ischemia resulting from ICA stenosis or thrombosis. The accelerated growth of the occipital lobe during fetal life heightens vascular demand, markedly affecting the ultimate configuration of the cranial width and potentially resulting in either an adult or fetal configuration from a transitional condition [30].

Morphological variations in PCA

Morphological changes or variations occur as a result of agenesis or involution during embryonic development [34]. Variations of the PCA include differences in the sources of origin, as well as variable sizes or structures of the PCA and its branches.

The term "fetal" or "fetal-type PCA" refers to the PCA, which initially arises from the ICA during embryonic development. When this embryonic pattern of origin of the PCA persists into adulthood, it is termed the “fetal-type PCA.” However, there is some disagreement among experts about whether certain variants should be classified as "fetal PCA” [35]. Some authors emphasize the importance of distinguishing between "true fetal" variants and those that are not [36]. This usually results in a hypoplastic or aplastic P1 segment and is commonly associated with a hypoplastic BA (capone). An adult-type PCA is defined as a configuration where the P1 segment of the PCA is larger than that of PComA, which is not hypoplastic [21].

Hypoplasia

**Figure 4:**
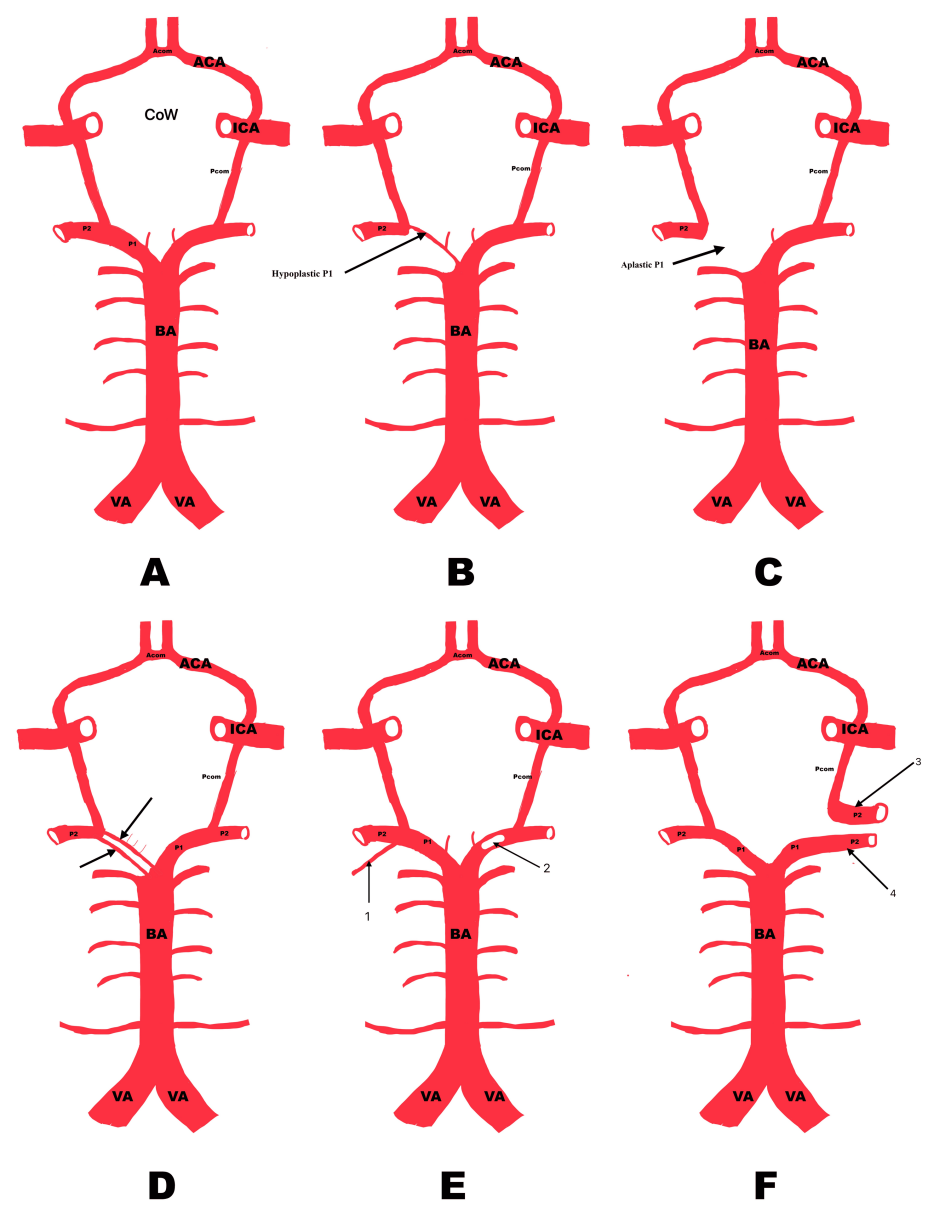
Morphological variations of the PCA or PCA segments PCA: posterior cerebral artery; ICA: internal carotid artery (A) complete circle of Willis, (B) arrow shows hypoplastic P1 segment, (C) arrow shows aplastic P1 segment, (D) arrows show duplication of P1 segment, (E) arrow 1 shows duplication of P2 segment arising from P1-P2 junction, and arrow 2 shows fenestration of P1 segment, (F) duplication of P2 segment: arrow 3 shows P2 segment arising from the ICA, and arrow 4 shows P2  segment arising from P1 segment Image credit: Dr. Neha Xalxo

In this condition, the P1 segment of the PCA is hypoplastic or smaller than PcomA, and the PCA primarily originates from the ICA through a prominent PcomA, and the main blood supply to the P2 arises from the ICA (Figure 4a) [5]. This is also referred to as fetal-type PCA (fPCA) (Figure 4B) [2].

Aplasia

It is an extremely rare scenario where the P1 segment may be absent, and PCA originates solely from the ICA. This is known as the "true fetal PCA" variant [1,7]. Occasionally, the PcomA continues as the PCA, referred to as fetal-type PCA (f-PCA), with a complete absence of the P1 segment. In some cases, the P2 segment may emerge directly from the ICA when the P1 segment is aplastic (Figure 4C) [37].

Duplication

**Figure 5:**
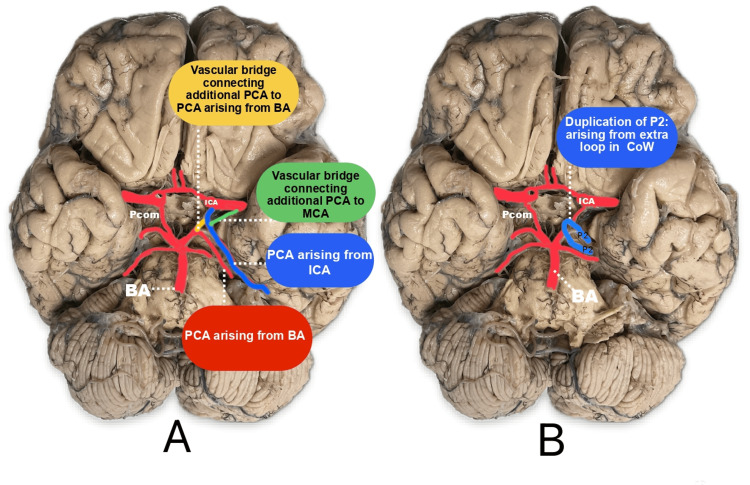
Morphological variations of the PCA or PCA segments PCA: posterior cerebral artery; ICA: internal carotid artery; MCA: middle cerebral artery; PcomA: posterior communicating artery (A) depicts an additional PCA that arises as a continuation of the ICA. A vascular bridge is formed between the PCA originating from the ICA and the PCA originating from the BA to A between the PCA originating from the ICA to the MCA. (B) depicts duplication of the P2 segment and its origin from the loop formed between PcomA and P1-P2 junction Image credit: Dr. Neha Xalxo

It is an anatomical variation where one branch arises from the bifurcation of the BA, and an additional branch may arise from the PcomA, P1, P2 segment, or the ICA [7,10,38,39]. Duplication typically occurs in the P1 segment, though it can also occur in the P2 segment (Figures 4D-4F). In rare cases, two separate P2 segments may arise from two distinct sources (Figure 5B) [21]. In another rare instance, a hyperplastic AchoA may continue as an additional PCA, supplying the PCA territory [40]. Additionally, in one case, the PCA may arise as a continuation of the ICA. A vascular bridge may form between the PCA originating from the ICA and the PCA originating from the BA. A vascular bridge may also connect the PCA originating from the ICA to the MCA (Figure 5A) [41].

Triplication

It is a rare occurrence when branches arise from the P1 segment with a smaller middle branch [7,41].

Fenestration

It is also called partial or incomplete duplication, in which a segment of a vessel splits into two channels before and then rejoins [42]. It is mostly observed in the P1 segment and can also be seen in the P2 segment or the distal PCA (Figure 4E) [43].

Variants of fPCA

Fetal-type PCA (fPCA) can be classified into various subtypes based on the relative size of the P1 segment and the PComA [44]:

(a) Partial fPCA: The P1 segment is smaller than the PComA [9,39,45].

(b) Intermediate (or transitional) fPCA: The P1 segment and PComA are equal in diameter [45,46]. This variant is also known as the adult-type PCA [21,44].

(c) Full fPCA: The P1 segment is absent, or the P1 segment is not visualized on computed tomography angiography (CTA) or magnetic resonance angiography (MRA) [39,45].

Clinical aspects of PCA variations

Morphological variations of the PCA can lead to ischemic stroke, aneurysms, and other cerebrovascular events. The fetal-type PCA, seen in 12%-36% of cases, may cause severe cognitive impairment in cases of ICA stenosis or embolism due to an increased risk of hypoperfusion [47]. Aneurysms of the P2 segment of PCA are rare but can have high mortality if ruptured. These aneurysms are often associated with complications related to the occipital lobe and visual pathways [2]. Infarctions in the PCA territory commonly present with visual disturbances, like contralateral homonymous hemianopia (often macular-sparing), and may also lead to sensory deficits, cognitive deficits, visual neglect, or aphasia, with left-sided infarcts often leading to alexia and visual agnosia, while right-sided infarcts may cause prosopagnosia and spatial disorientation. In rare cases, depending on the involvement of the lateral thalamus and midbrain, it may lead to hemiplegia [4,48]. Duplications in the PCA can disrupt blood flow and increase the risk of embolism, requiring tailored management approaches during cerebrovascular events [17]. The complexity of PCA variations significantly influences surgical planning. To avoid damaging critical structures, such as the oculomotor nerve or brainstem, during revascularization or aneurysm procedures, it is crucial to thoroughly understand the anatomy and its variations [9,49]. Understanding the morphology and anatomical variations of PCA is essential for improving patient outcomes in PCA-related cerebrovascular events. It will help neurosurgeons and radiologists accurately diagnose, plan appropriate interventions, or modify treatment strategies, ensuring better management of these conditions.

## Conclusions

This literature review studied the anatomy of the PCA, including its segmentation, branching patterns, and anomalies. Knowledge of the microsurgical anatomy of the PCA is important for the microneurosurgeon in order to deal with the various pathologies in this region without further complications. This information is also useful for planning and performing interventional procedures and surgeries within the posterior cranial fossa. Further research is essential with the help of cadaveric studies and noninvasive imaging procedures to elucidate the clinical significance of these variations, which will ultimately enhance both preventive and therapeutic approaches in managing cerebrovascular diseases.
